# Global research of artificial intelligence in strabismus: a bibliometric analysis

**DOI:** 10.3389/fmed.2023.1244007

**Published:** 2023-09-20

**Authors:** Ziying Zhou, Xuan Zhang, Xiajing Tang, Andrzej Grzybowski, Juan Ye, Lixia Lou

**Affiliations:** ^1^Eye Center, The Second Affiliated Hospital, School of Medicine, Zhejiang University, Zhejiang Provincial Key Laboratory of Ophthalmology, Zhejiang Provincial Clinical Research Center for Eye Diseases, Zhejiang Provincial Engineering Institute on Eye Diseases, Hangzhou, China; ^2^Institute for Research in Ophthalmology, Foundation for Ophthalmology Development, Poznan, Poland

**Keywords:** strabismus, artificial intelligence, bibliometric analysis, global publications, keywords analysis

## Abstract

**Purpose:**

To analyze the global publications on artificial intelligence (AI) in strabismus using a bibliometric approach.

**Methods:**

The Web of Science Core Collection (WoSCC) database was used to retrieve all of the publications on AI in strabismus from 2002 to 2023. We analyzed the publication and citation trend and identified highly-cited articles, prolific countries, institutions, authors and journals, relevant research domains and keywords. VOSviewer (software) and Bibliometrix (package) were used for data analysis and visualization.

**Results:**

By analyzing a total of 146 relevant publications, this study found an overall increasing trend in the number of annual publications and citations in the last decade. USA was the most productive country with the closest international cooperation. The top 3 research domains were Ophthalmology, Engineering Biomedical and Optics. Journal of AAPOS was the most productive journal in this field. The keywords analysis showed that “deep learning” and “machine learning” may be the hotspots in the future.

**Conclusion:**

In recent years, research on the application of AI in strabismus has made remarkable progress. The future trends will be toward optimized technology and algorithms. Our findings help researchers better understand the development of this field and provide valuable clues for future research directions.

## 1. Introduction

Strabismus, featured by misalignment of the eyes, is one of the most common ocular diseases (1). It can occur at all ages, while children are diagnosed more commonly, with a prevalence ranging from 2.40 to 5.65% (2–6). For young children, it is typical that strabismus occurs spontaneously, probably stemming from inheritance, low birth rate, prematurity and etc. (7). Individuals suffering from strabismus later in life may have gone through head injury, stroke, eye muscle damage during surgery or developed diseases, such as Graves’ disease, diabetes and myasthenia gravis (1, 8, 9). Traditionally, the diagnosis of strabismus requires for specialized examinations like the cover and uncover test, the Hirschberg test and the alternate prism cover test conducted by ophthalmologists (3, 10). In addition to resulting in amblyopia and irreversible vision loss, strabismus plays a significant role in developing mental illness and reducing quality of life (11–13). Therefore, conducting researches on strabismus is of great significance.

Known as the fourth industrial revolution in the history of humanity (14), artificial intelligence (AI) is an incredibly hot topic, referring to the area of computer science devoting to creating machine that can undertake behaviors that humans consider intelligent (15). In the field of medicine, AI has its unique advantages in medical imaging analysis (16). It is AI technology that enables computers and systems to obtain useful information from digital images, videos and other visual inputs and carry out analysis (17, 18). Since ocular images have vital clinical implications, ophthalmology has always been in a leading position in artificial intelligence and technological applications (19). And strabismus is a suitable field for the application of AI technology because a significant portion of investigations are image-based. A certain number of researches concerning automatic strabismus diagnosis, quantitative measurement and other relevant achievements have been reported.

Bibliometric analysis is a statistical method to exhibit previous research achievements and identify research hotspots by quantitatively assess the research status of countries, institutions, authors, journals, which cannot be replaced by other methods including traditional reviews, meta-analyses (20, 21). It has been applied in numerous subjects and disciplines, such as economics, agriculture, engineering, medicine and so forth (22–25). Bibliometrics has been commonly utilized in analyzing scientific publications on AI in ophthalmology, such as glaucoma, diabetic retinopathy and macular edema (26–28). However, no bibliometric analysis of AI in strabismus has been conducted before. Hence, this is the first study aiming to present an overview regarding the global publications of AI in strabismus and to provide a prediction of future research directions in this field.

## 2. Materials and methods

### 2.1. Data collection

Based on the recommendation of collecting bibliometric data from single database (20), we used Web of Science Core Collection (WoSCC) as the research database, which is the most commonly used and approved database for bibliometric analysis. To search for relevant data, at least one keyword related to strabismus and at least one keyword related to AI were combined to form query formulation. The detailed query formulation was described as follows: TS = (AI OR “artificial intelligence” OR intelligent OR “data learning” OR “robotic*” OR “computer vision” OR “machine learning” OR “deep learning” OR “deep network*” OR “neural learning” OR automat* OR algorithm OR “neural network*” OR “expert* system*”) AND TS = (strabismus OR esotropia OR exotropia OR hypertropia OR hypotropia OR heterotropia OR esophoria OR exophoria OR hyperphoria OR hypophoria OR heterophoria OR “dissociated vertical deviation” OR “dissociated horizontal deviation” OR “dissociated torsional deviation” OR “[(eye OR ocular) AND (motility OR movement*) AND disorder*]” OR “[(eye OR ocular) AND (alignment OR deviation)]” OR “media rectus” OR “lateral rectus” OR “superior rectus” OR “inferior rectus” OR “superior oblique” OR “inferior oblique” OR “third nerve” OR “fourth nerve” OR “sixth nerve” OR “abducens nerve” OR “oculomotor nerve” OR “trochlear nerve”). The time span was from January 1, 2002 to March 31, 2023, and the document types were limited to articles, reviews and proceedings papers. The last search was conducted on April 29, 2023. A total of 280 retrieved documents were prepared for the following screening.

### 2.2. Data screening

To exclude irrelevant documents in retrieved documents, we set the practical inclusion criteria as follows: (i) involvement of AI technology, including deep learning, machine learning and automated devices; (ii) involvement of strabismus, including: (1) researches focused on strabismus; (2) researches focused on the characterized clinical features of strabismus; (3) researches focused on multiple diseases and strabismus. After reading the titles and abstracts of each document carefully, 148 documents were included for the bibliometric analysis.

### 2.3. Data analysis and visualization

Publications and citations of each year, countries, institutions, authors, journals, research domain and H-index were acquired from WoSCC. H-index, as a reference index, indicates the impact of a researcher, country, institution, journal on the development of the certain scientific field (29, 30). The WoSCC intrinsic toolkits help to analyze general features mentioned above. Microsoft Excel 2019 was used to conduct polynomial regression analysis and to export charts and tables of publications, top-cited documents, productive countries, institutions, authors and journals, and hot research domain. The average growth rate of publications was calculated as follows:


$$\mathrm{Growth}\;\mathrm{rate}=\left(\sqrt[{t_{2}-t_{1}}]{p_{2}÷p_{1}}-1\right)\times 100$$

Where $t_{1}$ : first year; $t_{2}$ : last year; $p_{1}$ : publication count of the first year; $p_{2}$ : publication count of the last year.

We used VOSviewer (software, version 1.6.19) (31) to visualize the collaboration of countries, institutions, authors and co-occurrence of keywords, and calculate the total link strength. Bibliometrix (package, version 4.1.2) of R (programming language, version 4.3.0) (32) was applied to create WordCloud of keywords and calculate fractionalized frequency of an author which reflect one’s contribution to the publications. Fractionalized frequency was calculated as follows:


$$\mathrm{Frac}\;\mathrm{Freq}\left({\mathrm{AU}}_{j}\right)=\underset{h\in {\mathrm{AU}}_{j}}{\sum }\frac{1}{n.\mathrm{of}\;\mathrm{CoAuthors}\left(h\right)}$$

Where ${\mathrm{AU}}_{j}$ : the set of documents co-authored by the author *j*; *h*: a document included in ${\mathrm{AU}}_{j}$ .

## 3. Results

### 3.1. Analysis of publications and citations

**Figure 1:**
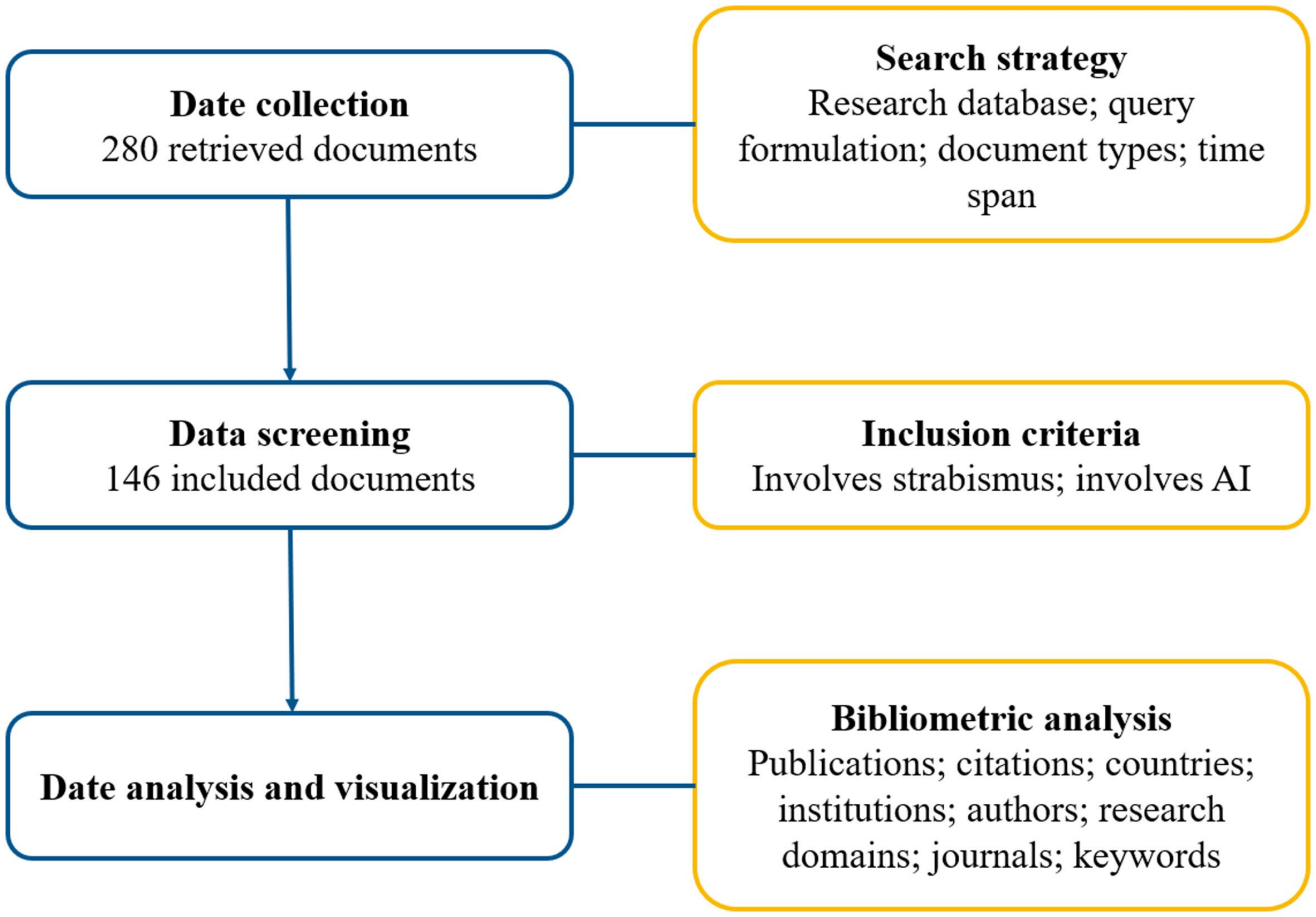
Detailed flowchart of this study.

On the basis of search strategy and inclusion criteria, we included 146 documents (Figure 1), including 110 articles, 26 proceedings and 10 reviews. The detailed publication numbers of different document types during 2002 and 2023 were shown in Supplementary Table S1. And Supplementary Figure S1 illustrated the annual trends of publications and citations on AI in strabismus from 2002 to 2023. From 2002 to 2012, the number of publications was no more than 5 documents per year, seeming to be relatively small. Since 2013, there has been an overall rising trend and at the year of 2021 the number of publications per year approached the peak value of 25. The average growth rate from 2002 to 2022 was 16.71%. Especially the last 5 years (2018–2022), the research has developed rapidly, contributing 59.12% (81/137) of all documents from 2002 to 2022. The total citations were up to 1,222 times, and average citations per item and H-index were 8.37 and 18, respectively. To model the publication and citation trends, polynomial regression analysis was conducted (2023 was excluded due to incomplete indexing). The fitted curves of *y*1 = −2E-05x^6^ + 0.0013x^5^–0.0332x^4^ + 0.3959x^3^ – 2.2868x^2^ + 5.8559x − 2.627(*R*^2^ = 0.8423) and *y*2 = −0.0003x^6^ + 0.0212x^5^ – 0.498x^4^ + 5.5629x^3^ – 29.853x^2^ + 70.569x − 50.265(*R*^2^ = 0.9642) indicated changes in the quantities of publications and citations with time, respectively. The trend of citations showed a similar tendency as publications, indicating AI in strabismus drew researchers’ attention in the last decade and is in the developing phase.

Additionally, we listed the top 10 documents ranked by annual citations count in Table 1. There were 7 articles, 2 reviews and a proceedings paper. Eight documents were published in the last 5 years. The earliest document, also the most impactful one, was by Donahue et al. (33). It drew up guidelines for automated preschool vision screening, including the identification of children with strabismus. The Pediatric Vision Scanner, detecting the absence of foveal fixation as a harbinger of strabismus and amblyopia, was regarded as a potentially recommended instrument. The top 10 documents ranked by total citations count were also listed in Table 1. Four articles and 1 proceedings paper published before 2014 were newly on the list.

**Table 1:** Top documents ranked by annual citations/total citations.

References	Title	Year	Source title	Annual citations	Document type
Donahue et al. (33)	Guidelines for automated preschool vision screening: a 10-year, evidence-based update	2013	Journal of AAPOS	16.27	Proceedings paper
Chen et al. (34)	Strabismus recognition using eye-tracking data and convolutional neural networks	2018	Journal of Healthcare Engineering	7.17	Article
Arnold et al. (35)	AAPOS uniform guidelines for instrument-based pediatric vision screen validation 2021	2022	Journal of AAPOS	6.50	Article
Chen et al. (36)	Eye-tracking-aided digital system for strabismus diagnosis	2018	Healthcare Technology Letters	5.33	Article
Pundlik et al. (37)	Development and preliminary evaluation of a smartphone app for measuring eye alignment	2019	Translational Vision Science & Technology	5.20	Article
Reid et al. (38)	Artificial intelligence for pediatric ophthalmology	2019	Current Opinion in Ophthalmology	4.80	Review
Miao et al. (39)	Virtual reality-based measurement of ocular deviation in strabismus	2020	Computer Methods and Programs in Biomedicine	4.50	Article
Yehezkel et al. (40)	Automated diagnosis and measurement of strabismus in children	2020	American Journal of Ophthalmology	3.50	Article
Ji et al. (41)	Eye and mouth state detection algorithm based on contour feature extraction	2018	Journal of Electronic Imaging	3.17	Article
Strianese (42)	Update on Graves disease: advances in treatment of mild, moderate and severe thyroid eye disease	2017	Current Opinion in Ophthalmology	3.14	Review
**References**	**Title**	**Year**	**Source title**	**Total citations**	**Document type**
Donahue et al. (33)	Guidelines for automated preschool vision screening: a 10-year, evidence-based update	2013	Journal of AAPOS	179	Proceedings paper
Ben Simon et al. (43)	Strabismus after deep lateral wall orbital decompression in thyroid-related orbitopathy patients using automated Hess screen	2006	Ophthalmology	51	Article
Schaeffel (44)	Kappa and Hirschberg ratio measured with an automated video gaze tracker	2002	Optometry and Vision Science	49	Article
Chen et al. (34)	Strabismus recognition using eye-tracking data and convolutional neural networks	2018	Journal of Healthcare Engineering	43	Article
Han et al. (45)	Quantification of heterophoria and phoria adaptation using an automated objective system compared to clinical methods	2010	Ophthalmic and Physiological Optics	40	Article
Chen et al. (36)	Eye-tracking-aided digital system for strabismus diagnosis	2018	Healthcare Technology Letters	32	Article
Hunter et al. (46)	Pediatric vision screener 1: instrument design and operation	2004	Journal of Biomedical Optics	30	Proceedings paper
Ransbarger et al. (47)	Results of a community vision-screening program using the Spot photoscreener	2013	Journal of AAPOS	27	Article
Pundlik et al. (37)	Development and preliminary evaluation of a smartphone app for measuring eye alignment	2019	Translational Vision Science & Technology	26	Article
Reid et al. (38)	Artificial intelligence for pediatric ophthalmology	2019	Current Opinion in Ophthalmology	24	Review

### 3.2. Analysis of top productive countries and collaboration networks of countries

A total of 28 countries had published related research on this topic. Countries ranked by publication count were listed in the Table 2. Accounting for 35.62% (52/146) of included documents, USA was the most productive country with the most publications and the highest citations (687 times) and H-index (15). According to publication count, China (29/146, 19.86%) was the second leading country, followed by South Korea (11/146, 7.53%). When it came to collaboration networks of countries, we visualized it by means of VOSviewer (Supplementary Figure S2). The number of publications determined the size of the circle and the lines between circles stood for the co-authorship between countries. The thickness of lines indicated the strength of cooperation (termed total link strength). USA had the highest total link strength, followed by England and China. Seventeen countries with cooperative relationships were presented, while 11 countries lacking international cooperation were not shown in Supplementary Figure S2, like South Korea, Brazil etc.

**Table 2:** Top countries/institutions ranked by publication count.

Country	Documents	%	Citations	Citation per item	H-index	Total link strength*
USA	52	35.62	687	13.21	15	14
China	29	19.86	180	6.21	6	9
South Korea	11	7.53	63	5.73	4	0
England	10	6.85	45	4.50	4	10
Brazil	9	6.16	59	6.56	3	0
India	9	6.16	35	3.89	3	1
Germany	8	5.48	84	10.50	4	4
Israel	6	4.11	79	13.17	4	4
Switzerland	6	4.11	17	2.83	2	3
France	5	3.42	58	11.60	3	4
**Institution (country)**	**Documents**	**%**	**Citations**	**Citation per item**	**H-index**	**Total link strength**^#^
Johns Hopkins University (USA)	11	7.53	127	11.55	8	8
Vanderbilt University (USA)	6	4.11	203	33.83	4	13
Universidade Federal do Maranhao (Brazil)	6	4.11	54	9.00	3	4
Seoul National University (South Korea)	6	4.11	40	6.67	3	7
Chu Hai College of Higher Education (China)	5	3.42	93	18.60	4	9
The Hong Kong Polytechnic University (China)	4	2.74	89	22.25	4	7
Harvard Medical School (USA)	4	2.74	31	7.75	5	14
University of California, Los Angeles (USA)	3	2.05	65	21.67	2	9
Harvard University (USA)	3	2.05	53	17.67	5	1
University of Pennsylvania (USA)	3	2.05	31	10.33	2	8

^*^The total link strength was analyzed from all countries using VOSviewer software.

^#^The total link strength was analyzed from all institutions using VOSviewer software.

### 3.3. Analysis of top productive institutions and collaboration networks of institutions

**Figure 2:**
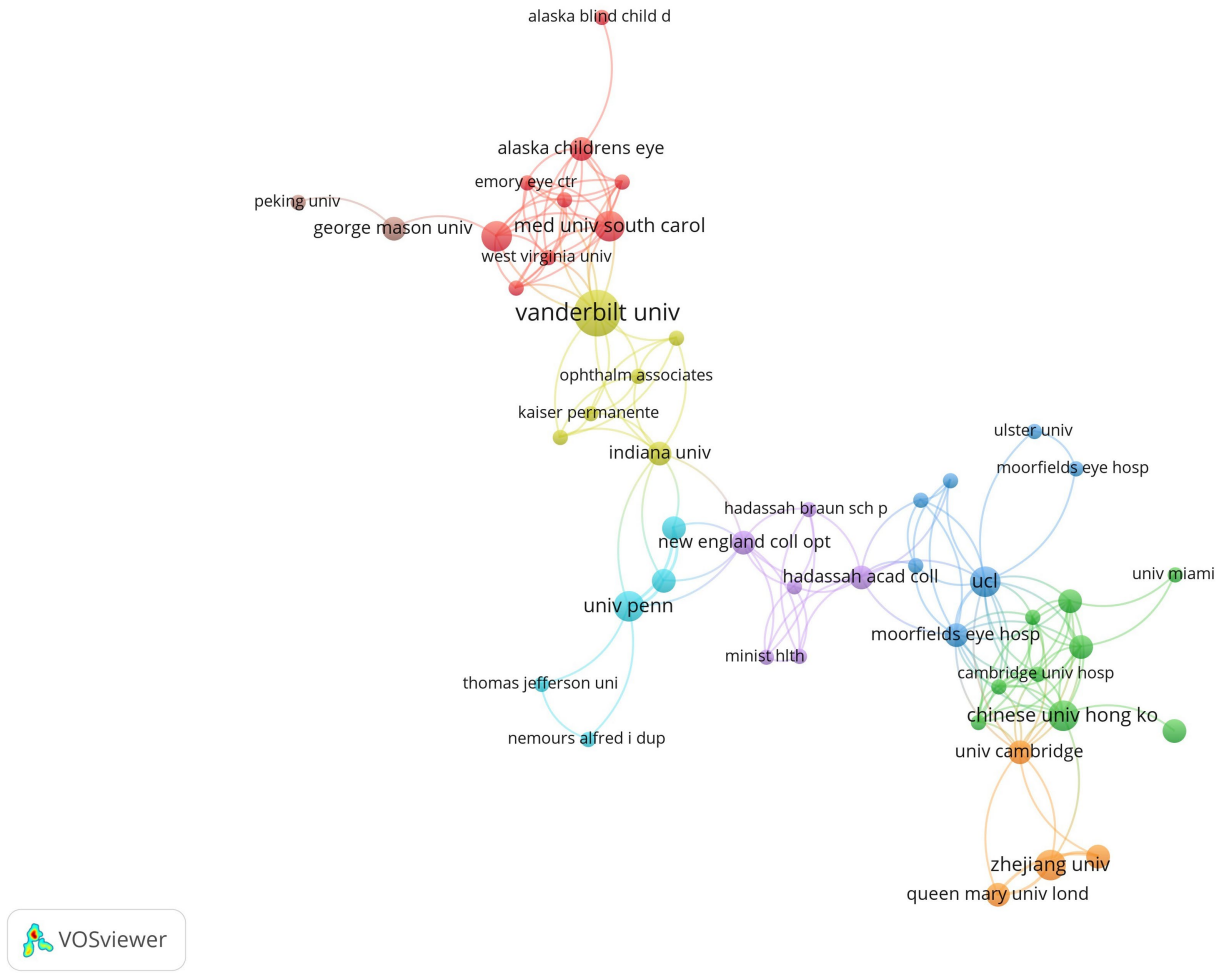
Co-authorship network visualization map of institutions. Each node represents one institution. The number of publications determines the size of the circle. Connecting lines represent collaboration between institutions.

There were 248 institutions participating in related research. Table 2 summarized institutions ranked by publication count. Johns Hopkins University had the greatest contribution with 11 documents in total, followed by Vanderbilt University, Universidade Federal do Maranhao and Seoul National University, each of which published 6 documents. Although the number of documents was inferior to Johns Hopkins University, Vanderbilt University had the highest citations. Figure 2 visualized the co-authorship network of institutions. While some institutions were not connected to each other, Figure 2 only demonstrated the largest set of connected items consisting of 48 items. University College London had the highest total link strength, followed by Moorfields Eye Hospital and Harvard Medical School. Nevertheless, the majority of institutions were dispersive and lack of cooperation (Supplementary Figure S3).

### 3.4. Analysis of top productive authors and collaboration networks of authors

**Figure 3:**
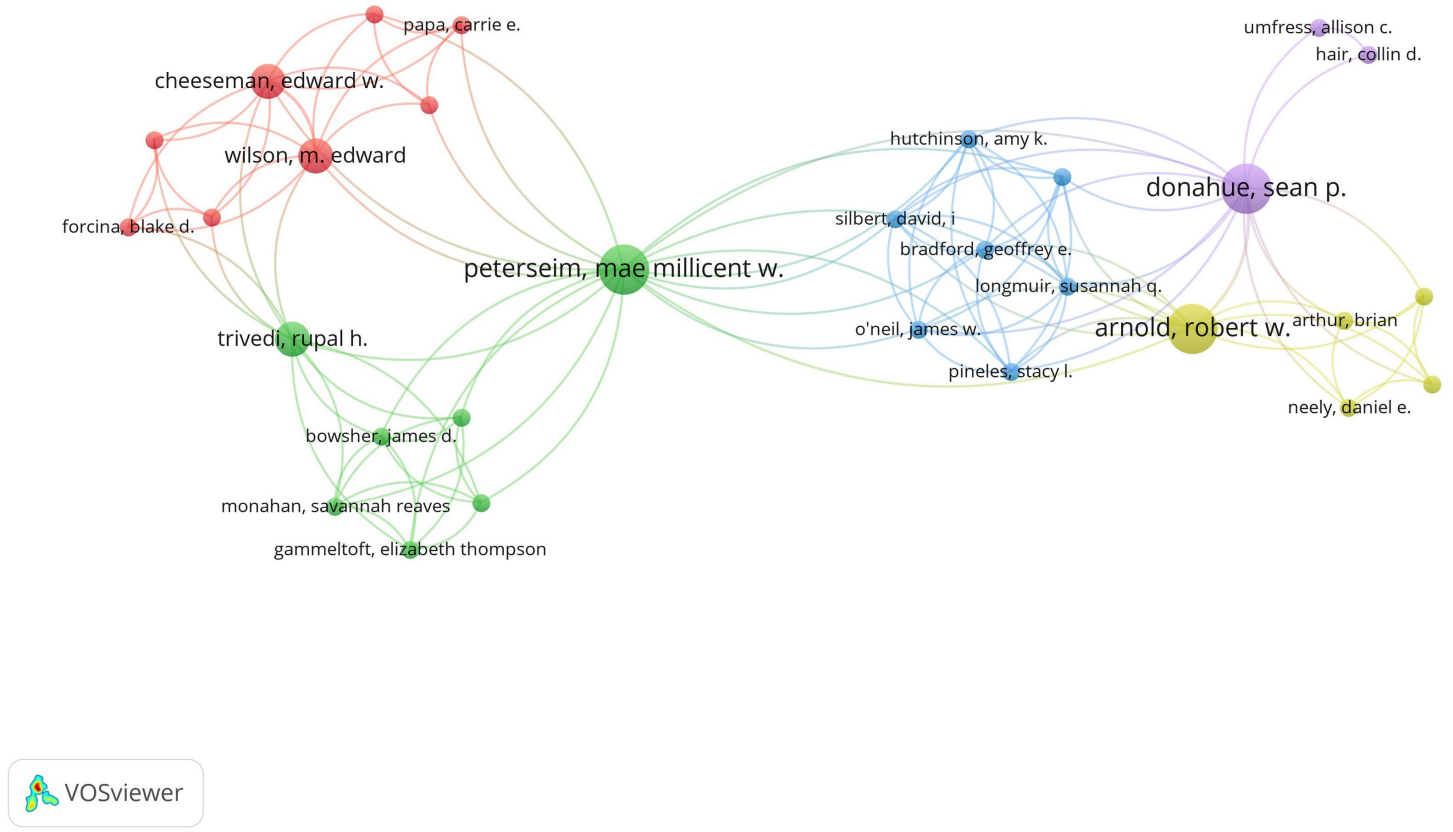
Co-authorship network visualization map of authors. Each node represents one author. The number of publications determines the size of the circle. Connecting lines represent collaboration between authors.

Top productive authors were listed in Table 3 and the collaboration relationship among authors were displayed in Figure 3. Because some authors were not connected to each other, the largest set of connected items consisting of 30 authors were visualized. According to Figure 3, we could see that authors from the same country were more likely to have close cooperation.

**Table 3:** Top authors ranked by publication count.

Author	Country	Publications	%	Citations	Citation per Item	H-index	Articles Fractionalized
Boris I. Gramatikov	USA	8	5.48	98	12.25	6	3.42
David L. Guyton	USA	8	5.48	96	12.00	6	1.87
Jo $\tilde{a}$ o Dallyson Sousa de Almeida	Brazil	7	4.79	56	8.00	3	1.27
Jorge Antonio Meireles Teixeira	Brazil	7	4.79	56	8.00	3	1.27
Aristófanes Correa Silva	Brazil	6	4.11	54	9.00	3	1.10
Jeong Min Hwang	South Korea	6	4.11	40	6.67	3	1.39
Hee Kyung Yang	South Korea	6	4.11	21	3.50	3	1.39
David G. Hunter	USA	5	3.42	78	15.60	4	1.00
Kristina Irsch	USA	5	3.42	36	7.20	3	1.25
Yikai Wu	China	5	3.42	36	7.20	3	1.25

### 3.5. Analysis of research domains and top productive journals

The 10 most common research domains were listed in Table 4. The top 3 research domains were Ophthalmology (59/146, 40.41%), Engineering Biomedical (21/146, 14.38%), and Optics (12/146, 8.22%).

**Table 4:** The most related research domains ranked by publication count.

Research domain (WoS categories)	Count	%
Ophthalmology	59	40.41
Engineering, Biomedical	21	14.38
Optics	12	8.22
Radiology, Nuclear Medicine and Medical Imaging	10	6.85
Computer Science, Artificial Intelligence	9	6.16
Engineering, Electrical and Electronic	9	6.16
Health Care Sciences and Services	9	6.16
Computer Science, Interdisciplinary Applications	8	5.48
Medical Informatics	8	5.48
Pediatrics	8	5.48

Top 10 productive journals were listed in Table 5. To evaluate the academic influence of journals, we consulted impact factor (IF) and the Journal Citation Reports (JCR) (2021) assessment system. Among these journals, JAMA Ophthalmology had the highest IF with 8.253. Journal of AAPOS (IF: 1.325, Q4) had the most publications and highest citations. Four journals ranked Q1 in JCR (2021), three journals ranked Q2, two journals ranked Q3 and one journal ranked Q4.

**Table 5:** Most productive journals ranked by publication count.

Source title	Count	%	Citations	H-index	IF (2021)	JCR (2021)
Journal Of AAPOS	6	4.11	248	5	1.325	Q4
Translational Vision Science Technology	5	3.42	48	4	3.048	Q2
Optometry and Vision Science	4	2.74	72	3	2.106	Q3
Investigative Ophthalmology Visual Science	4	2.74	37	3	4.925	Q1
Computers in Biology and Medicine	4	2.74	36	2	6.698	Q1
American Journal of Ophthalmology	4	2.74	27	3	5.488	Q1
Journal of Healthcare Engineering	3	2.05	46	2	3.822	Q2
JAMA Ophthalmology	3	2.05	40	3	8.253	Q1
Biomedical Engineering Online	3	2.05	31	3	3.903	Q3
PLoS One	3	2.05	26	3	3.752	Q2

### 3.6. Analysis of keywords

**Figure 4:**
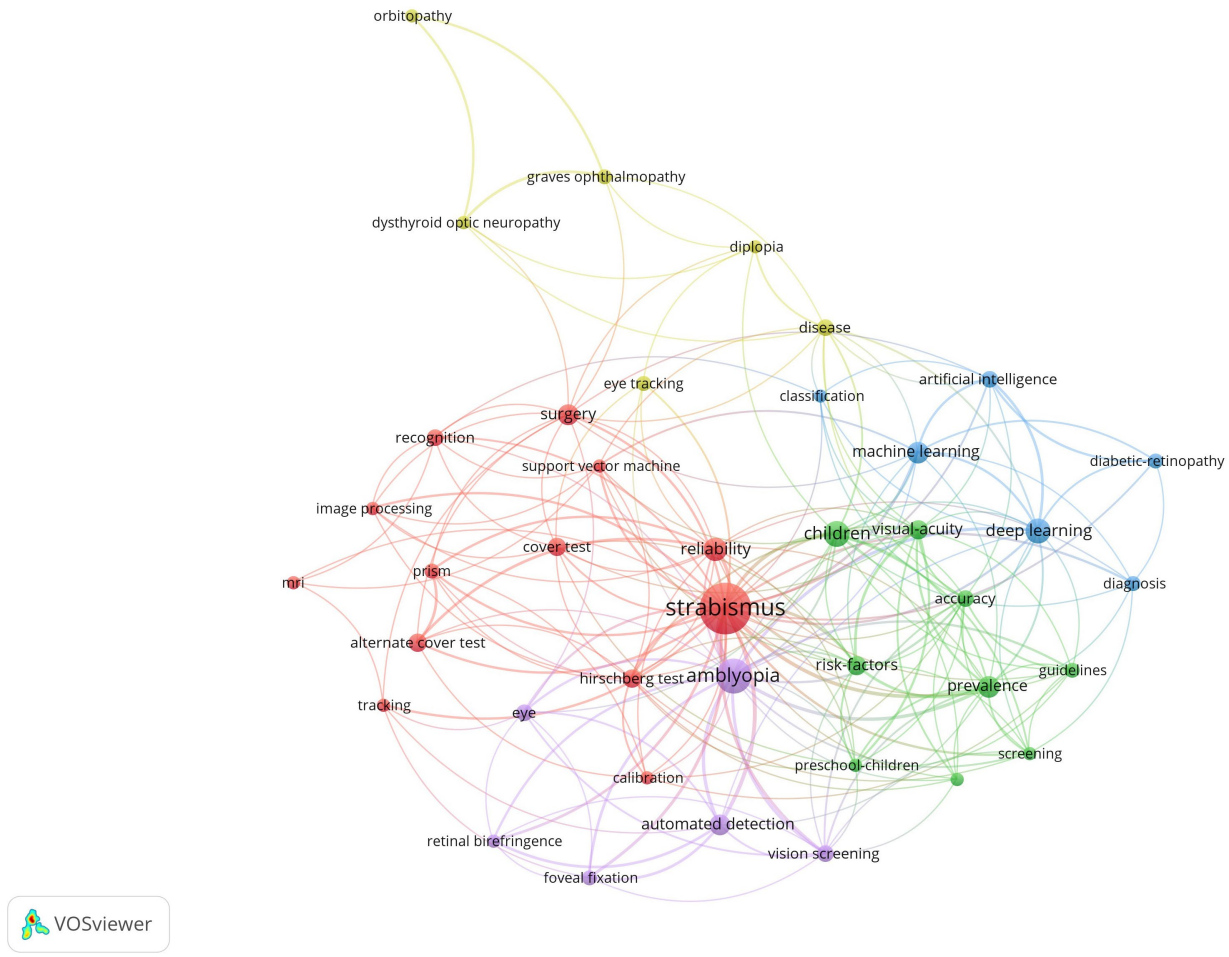
The co-occurrence map of keywords. It reveals 5 clusters (in 5 colors): the diagnosis of strabismus (in red), diseases related to strabismus (in yellow), terms related to AI (in blue), terms related to epidemiology (in green) and technical key point (in purple). Each node represents one keyword. The number of occurrences determines the size of the circle. Connecting lines represent co-occurrence between different keywords.

In order to have a better understanding of research orientation and prospect, we conducted a keyword co-occurrence analysis by VOSviewer. For the total of 663 automatically identified keywords, 40 keywords occurred at least 4 times, which were displayed in Figure 4. All included keywords were divided into 5 clusters, indicated by red, yellow, blue, green, and purple colors, representing the diagnosis of strabismus (e.g., “cover test”, “hirschberg test”, “recognition”, etc.), diseases related to strabismus (e.g., “graves ophthalmopathy”, “diplopia”, etc.), terms related to AI (e.g., “machine learning”, “deep learning”, etc.), terms related to epidemiology (e.g., “children”, “prevalence”, “risk factors”, etc.), and technical key point (e.g., “automated detection”, “vision screening”, etc.).

To understand when these hotspots emerged and how they evolved, we divided the documents into 3 group based on publication count and publication time: (i) 2002–2012 (during this period the academic output was relatively small, so time interval was extended); (ii) 2013–2017; (iii) 2018–2023. Three corresponding WordCloud of keywords (Supplementary Figure S4) were conducted by Bibiometrix Package in R. The more frequently-occurred, the bigger scale. “Strabismus” was the most dominant keyword for the entire period, followed by “amblyopia”. During 2002 and 2012, “polarization optics”, “vision screening” was ascendent. During 2013 and 2017, “retina birefringence” and “fixation detection” were more prevailing compared with “artificial neural networks”. During 2018 and 2023, “deep learning” and “machine learning” were dominant.

## 4. Discussion

In this study, we performed a bibliometric analysis to explore the features of the publications concerning the application of AI in strabismus from 2002 to 2023 and gave a comprehensive look at this research trends for the first time.

The production and growth rates of the scientific papers reflect how a particular subject of study is progressing. There was a relatively slow stage of development from 2002 to 2012. The number of publications during this period accounted for 18.49% (27/146) of the total. Then, there has been an overall rising tendency since 2013. Especially the last 5 years (2018–2022), the research has developed rapidly, contributing 59.12% (81/137) of all articles from 2002 to 2022. The advancements in AI technology and its use in ophthalmology were primarily the reason for the developing trend. The most influential document was guideline for automated preschool vision screening by Donahue et al. (33). Its high citations were closely related to its guiding value. And other impactful documents were focused on aspects including strabismus recognition using eye-tracking data and convolutional neural networks, instrument-based vision screen and eye alignment measurement using smartphone app (34–37, 39, 40, 44, 47).

USA, China and South Korea were the main forces in researches concerning the application of AI in strabismus. USA was the most productive country with the most publications and the highest citations and H-index. A significant gap between the number of publications of USA and China was noticed, while China ranked second in the number of publications. This was intimately tied to the fact that USA had the most advanced research equipment and a large number of scientific researchers in the whole world. Meanwhile, USA had the closest international cooperation, while other countries such as South Korea and Brazil sustained high scientific productions with poor international collaboration. Publications from USA in recent years inclined to study new screening devices which were able to detect amblyopia risk factors or directly detect strabismus (48, 49). Publications from China brought more focus on deep learning-based image analysis. Zhang et al. (50) proposed multi-feature fusion model which achieved an accuracy of 97.17%, sensitivity of 96.06%, specificity of 97.79%, and AUC of 0.969 in detecting strabismus. Shi and Tang (51) proposed a multitask deep learning model based on deep snake to improve strabismus iris recognition in complicated scenes. Publications from South Korea also paid more attention to automated detection of strabismus (52, 53).

Johns Hopkins University had excellent academic reputation and played a dominant role in this field. Research groups from here developed and reported a pediatric vision screener which was an efficient tool for detecting central fixation based on retinal birefringence scanning, and Gramatikov and Guyton did dedicate themselves to this research direction and exert great influence (54, 55). Researchers from Vanderbilt University which had the highest citations among all institutions, sought to a novel automated method of CT metrics of extraocular muscle in thyroid eye disease (56, 57) and made contribution to automated vision screening guidelines (33, 35). However, the majority of institutions were dispersive and lack of cooperation. It is crucial that the cooperation among countries and institutions needs to be reinforced. Authors from Brazil, including Jo $\tilde{a}$ o Dallyson Sousa de Almeida, Jorge Antonio Meireles Teixeira and Aristófanes Correa Silva, devoted themselves to researching the diagnosis of strabismus based on digital videos and surgical planning for horizontal strabismus by Support Vector Regression (58–60).

The top 3 research domains were Ophthalmology, Engineering Biomedical and Optics. Journal of AAPOS was ranked the first in the most productive journal. By analyzing keywords, “deep learning” and “machine learning” may be the hotspots in the near future. Meanwhile, “screening” and “smartphone app” had been the common interests of researchers. This may be due to the fact that the diagnosis of strabismus was important in clinical procedure and it was image-dependent which was suitable for AI technology to handle. AI-based algorithms can extract characteristics from ocular images to construct models to facilitate diagnosis and quantitively assessment.

By analyzing recent publications, the latest advances were manifested in different ways. When it came to screening devices, Monahan et al. (49) held the view that the blinq^™^ Vision Screener was suitable for detecting strabismus in children with sensitivity of 87.5% and specificity of 51.3%. As for new algorithms, Huang et al. (61) proposed a method that could automatically identify the oculomotor nerve from dMRI tractography and Xie et al. (62) put forward a novel multimodal deep-learning-based multi-class network for automated cranial nerves tract segmentation called CNTSeg, which would be beneficial to the diagnosis of incomitant strabismus. Lou et al. (63) proposed a novel deep learning-based approach to automatically evaluate the amount of inferior oblique overaction from frontal facial images via GAR2U-Net.

With the prosperity of AI technology, the development direction is changing from digital devices [like photoscreener, eye trackers, virtual reality headsets (39, 44, 64)] to automatic screening methods, which is more economical and practical in undeveloped areas. In the future, optimizing algorithms and proposing new techniques will become the focus of this field. For one thing, with the development of instrument-based vision screening technology, promoting the application of efficient and economical devices in strabismus screening is an important topic. For another, as artificial intelligence is racing ahead, it is vital to develop segmentation algorithms with higher accuracy and models with better performance. Also, an automated integrated platform with functions such as diagnosis, evaluation and postoperative prediction is highly expected.

Compared with AI applications in diabetic retinopathy, glaucoma and other eye diseases, AI applications in strabismus was relatively fewer. It may be because of the following reasons. On the one hand, the dynamic tests are required during the diagnosis of strabismus while a static fundus photograph is of great value for the diagnosis of eye diseases like diabetic retinopathy. On the other hand, public large-scale datasets of strabismus are rare, which is inconvenient for academic groups worldwide to test their models and algorithms. As a result, it is essential to grasp the specific characteristics of strabismus and promote the establishment of public large-scale datasets so as to provide opportunity for researchers worldwide to train their models. Research on the application of AI in strabismus has made significant progress in recent years, but there is still a distance between the current AI in strabismus and the clinical practice. We anticipate a more effective use of artificial intelligence in strabismus.

There are several limitations of the study. First, we only retrieve data from WoSCC. Other databases like Pubmed, Embase, and Scopus were not obtained. Secondly, our query formulation may not be perfect enough to retrieve all publications in the research field. Third, when analyzing keywords, similar keywords like “image processing” and “image processing techniques” were not merged.

## 5. Conclusion

This study presented an overview regarding the global researches of AI in strabismus. It was the first bibliometric analysis of this field. Our findings help researchers better understand the development of this field and provide valuable clues for future research directions. Research on the application of AI in strabismus has made remarkable progress in recent years. The future trends will be toward optimized technology and algorithms.

## Data availability statement

The original contributions presented in the study are included in the article/Supplementary material, further inquiries can be directed to the corresponding authors.

## Author contributions

ZZ, XZ, XT, JY, and LL: conception and design. JY and LL: administrative support. ZZ and LL: collection and assembly of data. ZZ, XZ, AG, and LL: data analysis and interpretation. All authors contributed to the article and approved the submitted version.

## Funding

This work was supported by National Natural Science Foundation of China [No. 82000948 to LL, No. U20A20386 to JY, No. 81870635 to JY]; National Key Research and Development Program of China [No. 2019YFC0118400 to JY]; and Zhejiang Provincial Key Research and Development Plan [No. 2019C03020 to JY].

## Conflict of interest

The authors declare that the research was conducted in the absence of any commercial or financial relationships that could be construed as a potential conflict of interest.

## Publisher’s note

All claims expressed in this article are solely those of the authors and do not necessarily represent those of their affiliated organizations, or those of the publisher, the editors and the reviewers. Any product that may be evaluated in this article, or claim that may be made by its manufacturer, is not guaranteed or endorsed by the publisher.
