# Supplementary material for: Global research of artificial intelligence in strabismus: a bibliometric analysis
Source: Front Med (Lausanne). 2023 Sep 20;10:1244007. doi: 10.3389/fmed.2023.1244007 (PMC10548140; doi:10.3389/fmed.2023.1244007)
Supplement: Supplementary file 1 [file Data_Sheet_1.docx]

Supplementary Material

Global research of artificial intelligence in strabismus: a bibliometric analysis

Ziying Zhou, Xuan Zhang, Xiajing Tang, Andrzej Grzybowski, Juan Ye*, Lixia Lou*

*** Correspondence:** Lixia Lou: loulixia110@zju.edu.cn, Juan Ye: yejuan@zju.edu.cn

# Supplementary Figures and Tables

## Supplementary Figures


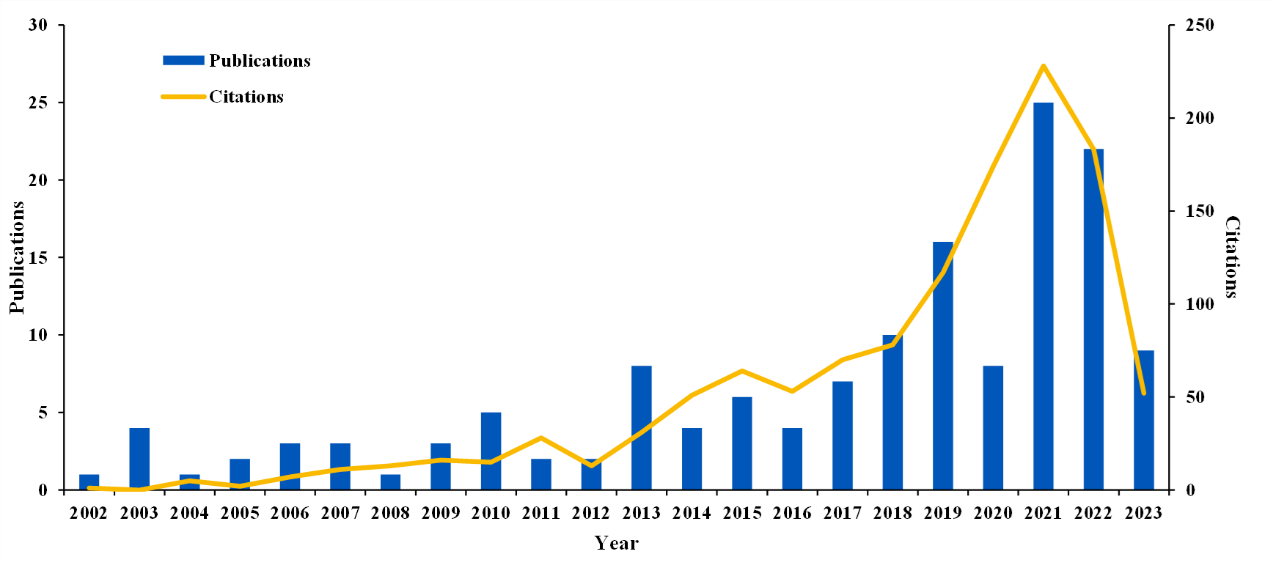


**Supplementary Figure S1.** Trend analysis of publications and citations.


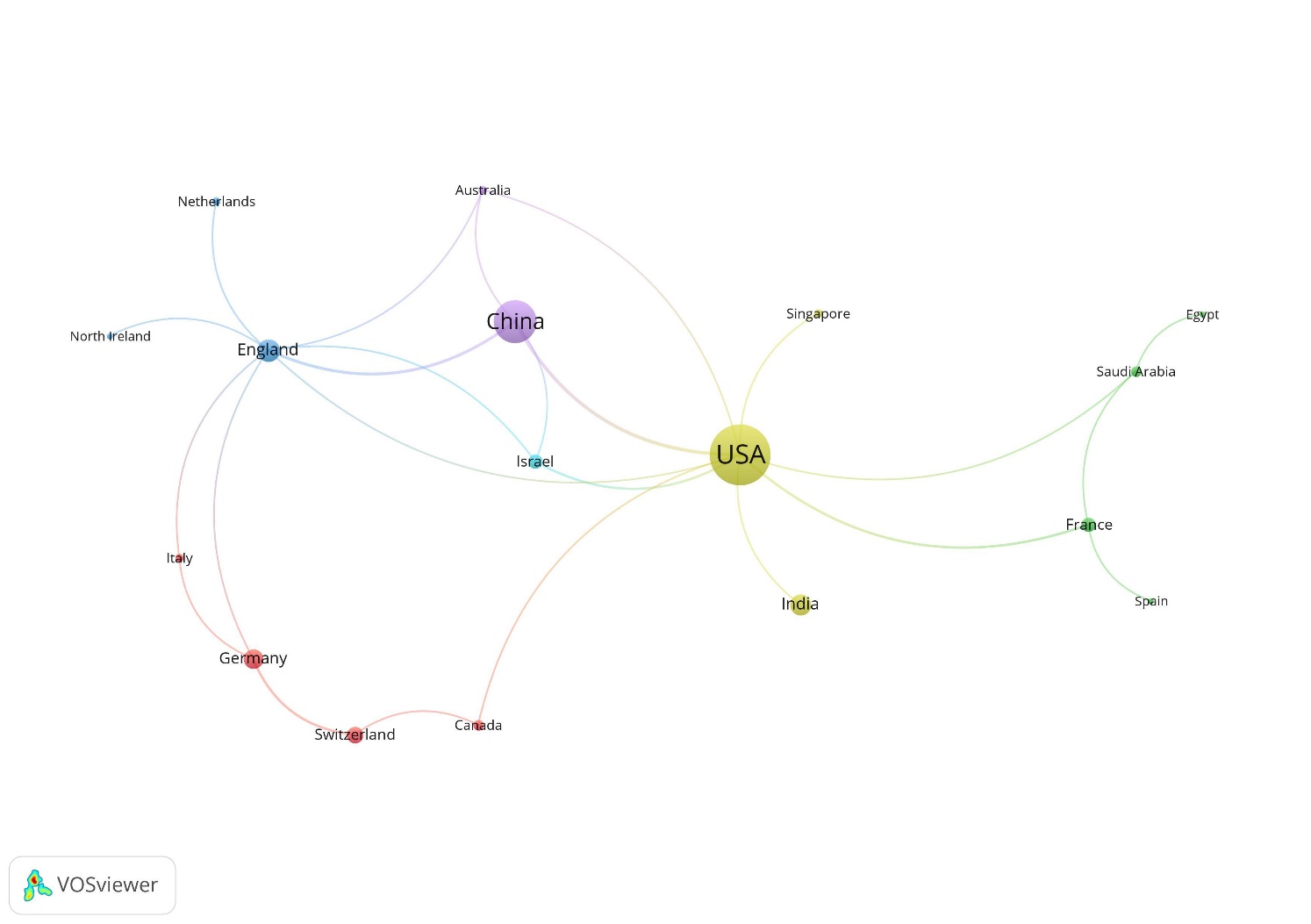


**Supplementary Figure S2.** Collaboration maps between countries. Each node represents one country. The number of publications determines the size of the circle. Connecting lines represent collaboration between countries.


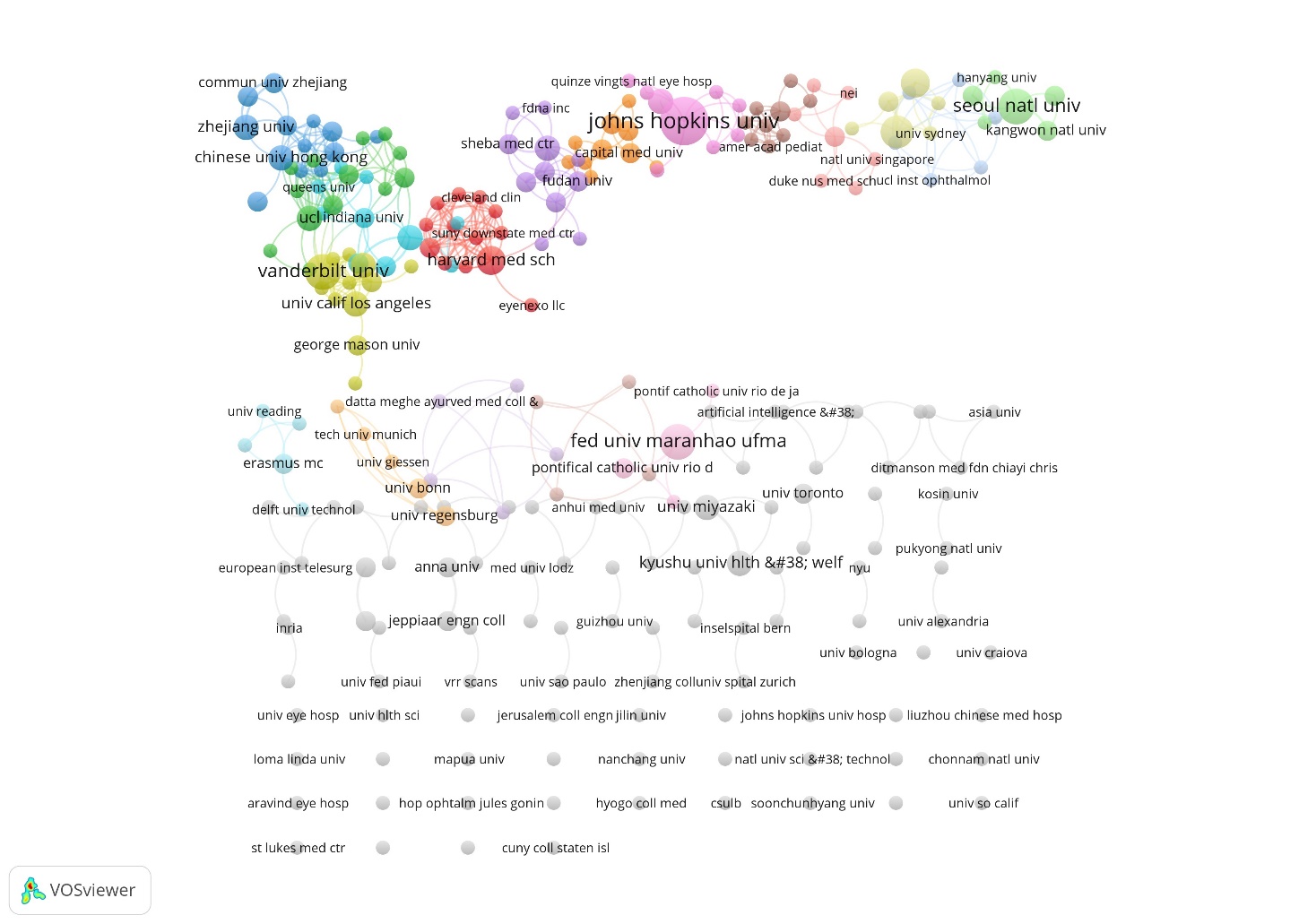


**Supplementary Figure S3.** Co-authorship network visualization map of 248 institutions. Each node represents one institution. The number of publications determines the size of the circle. Connecting lines represent collaboration between institutions.

**(A)**
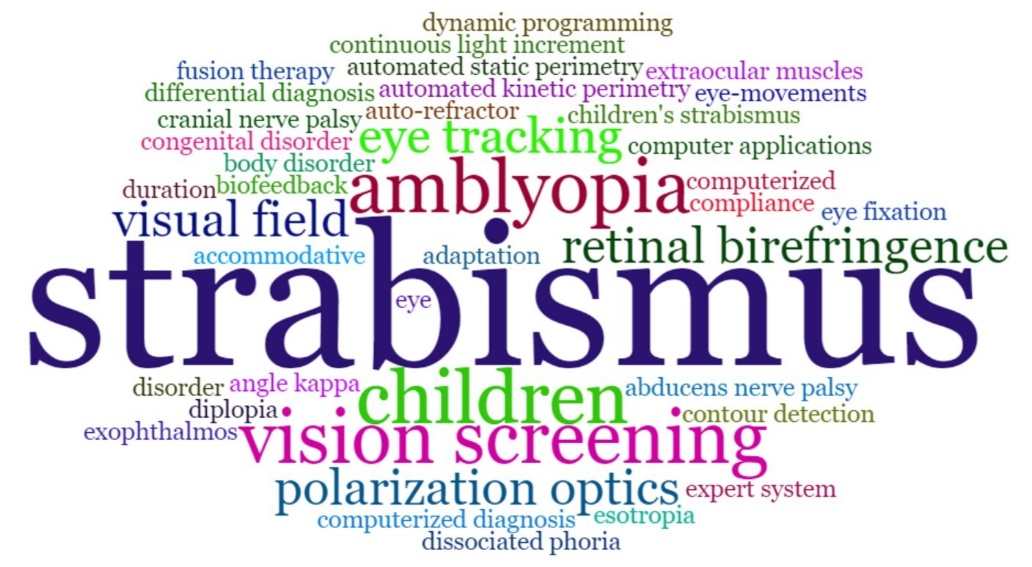


**(B)**
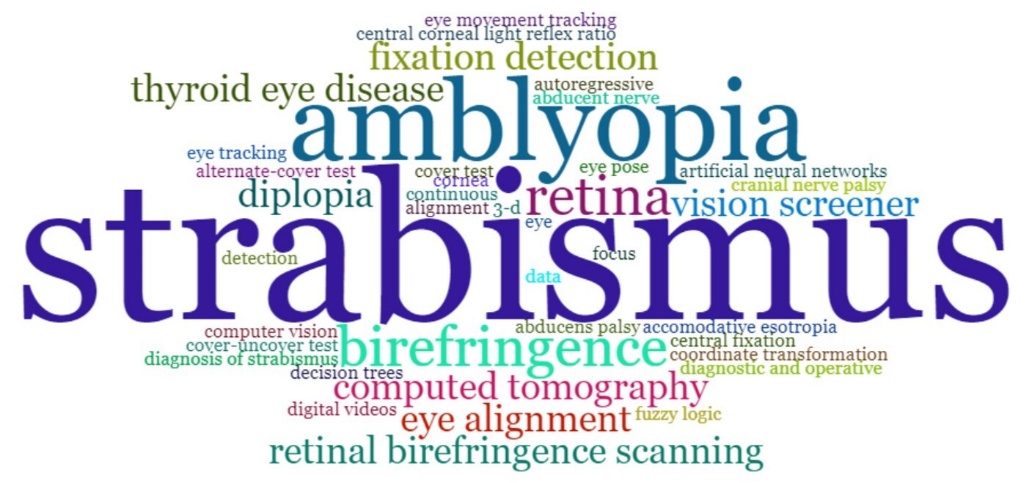


**(C)**
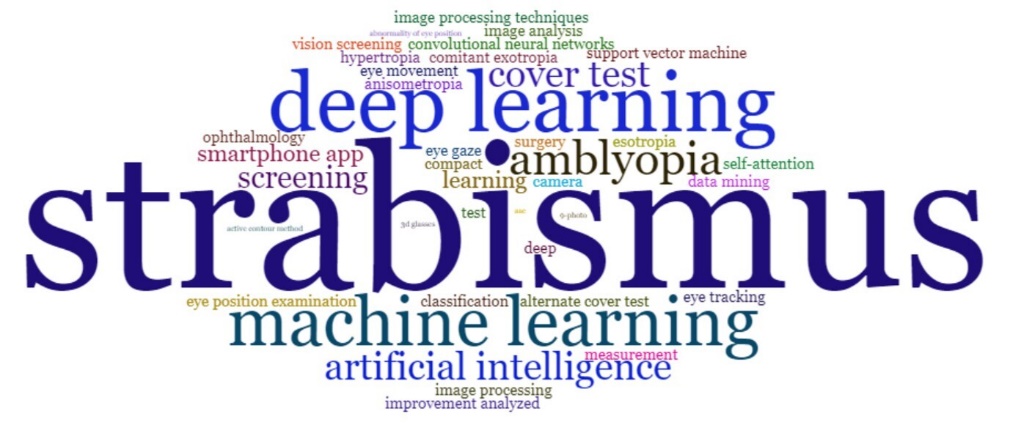


**Supplementary Figure S4.** WordCloud in 3 periods: **(A)** during 2002 and 2012; **(B)** during 2013 and 2017; **(C)** during 2018 and 2023. The number of occurrences determines the size of the keyword.

## Supplementary Tables

**Supplementary Table S1.** The annual publication count.

| Year | Document Type | | | Count |
| --- | --- | --- | --- | --- |
|  | **Articles** | **Proceedings** | **Reviews** |  |
| 2002 | 1 | 0 | 0 | 1 |
| 2003 | 1 | 3 | 0 | 4 |
| 2004 | 0 | 1 | 0 | 1 |
| 2005 | 2 | 0 | 0 | 2 |
| 2006 | 2 | 1 | 0 | 3 |
| 2007 | 2 | 0 | 1 | 3 |
| 2008 | 1 | 0 | 0 | 1 |
| 2009 | 1 | 2 | 0 | 3 |
| 2010 | 5 | 0 | 0 | 5 |
| 2011 | 2 | 0 | 0 | 2 |
| 2012 | 2 | 0 | 0 | 2 |
| 2013 | 4 | 4 | 0 | 8 |
| 2014 | 4 | 0 | 0 | 4 |
| 2015 | 4 | 2 | 0 | 6 |
| 2016 | 1 | 3 | 0 | 4 |
| 2017 | 4 | 1 | 2 | 7 |
| 2018 | 9 | 1 | 0 | 10 |
| 2019 | 11 | 3 | 2 | 16 |
| 2020 | 7 | 1 | 0 | 8 |
| 2021 | 19 | 3 | 3 | 25 |
| 2022 | 20 | 1 | 1 | 22 |
| 2023 | 8 | 0 | 1 | 9 |
| total | 110 | 26 | 10 | 146 |
